# Biophysical and enzymatic properties of the simian and prototype foamy virus reverse transcriptases

**DOI:** 10.1186/1742-4690-7-5

**Published:** 2010-01-29

**Authors:** Maximilian J Hartl, Florian Mayr, Axel Rethwilm, Birgitta M Wöhrl

**Affiliations:** 1 Universität Bayreuth, Lehrstuhl für Struktur und Chemie der Biopolymere & Research, Center for Biomacromolecules, 95440 Bayreuth, Germany; 2 Universität Würzburg, Institut für Virologie und Immunbiologie, 97078 Würzburg, Germany

## Abstract

**Background:**

The foamy virus Pol protein is translated independently from Gag using a separate mRNA. Thus, in contrast to *orthoretroviruses *no Gag-Pol precursor protein is synthesized. Only the integrase domain is cleaved off from Pol resulting in a mature reverse transcriptase harboring the protease domain at the N-terminus (PR-RT). Although the homology between the PR-RTs from simian foamy virus from macaques (SFVmac) and the prototype foamy virus (PFV), probably originating from chimpanzee, exceeds 90%, several differences in the biophysical and biochemical properties of the two enzymes have been reported (i.e. SFVmac develops resistance to the nucleoside inhibitor azidothymidine (AZT) whereas PFV remains AZT sensitive even if the resistance mutations from SFVmac PR-RT are introduced into the PFV PR-RT gene). Moreover, contradictory data on the monomer/dimer status of the foamy virus protease have been published.

**Results:**

We set out to purify and directly compare the monomer/dimer status and the enzymatic behavior of the two wild type PR-RT enzymes from SFVmac and PFV in order to get a better understanding of the protein and enzyme functions. We determined kinetic parameters for the two enzymes, and we show that PFV PR-RT is also a monomeric protein.

**Conclusions:**

Our data show that the PR-RTs from SFV and PFV are monomeric proteins with similar biochemical and biophysical properties that are in some aspects comparable with MLV RT, but differ from those of HIV-1 RT. These differences might be due to the different conditions the viruses are confronted with in dividing and non-dividing cells.

## Background

Foamy viruses (FVs) belong to the family *retroviridae*, but differ in several aspects from *orthoretrovirinae*: (a) reverse transcription occurs before the virus leaves the host cell [1,2], (b) the *pol*-gene is expressed from a separate mRNA [3-5], and (c) the viral protease is not cleaved off from the Pol polyprotein. Only the integrase is removed from Pol [6,7]. Thus, the FV reverse transcriptase harbors a protease, polymerase and RNase H domain (PR-RT) (for review see [8,9]).

Only recently, studies have focused on the biochemistry of the PR-RTs of FVs. Although the PR-RTs from simian foamy virus from macaques (SFVmac) and from the prototype foamy virus (PFV) exhibit more than 90% sequence homology at the protein level (79.5% identity; LALIGN, http://www.ch.embnet.org), some differences in their behavior have been reported. Bacterially expressed PFV PR-RT harbors many characteristics of orthoretroviral RTs; however, FV enzymes exhibit some peculiar features [10-16]. In comparison to human immunodeficiency virus type 1 (HIV-1) RT, PFV PR-RT appears to be a more processive polymerase [11]. This is probably due to differences in virus assembly. FV Pol packaging has been reported to require interactions of Pol with specific sequences in the RNA genome [17], and it has been suggested that there is a lower number of FV Pol molecules in the virus particle as compared to orthoretroviruses [11]. As a consequence, a highly processive polymerase is essential to enable synthesis of the complete double stranded genome.

One antiretroviral drug that has been shown to inhibit FV replication is azidothymidine (AZT) [1,18,19]. In *in vivo *experiments SFVmac acquired high resistance to AZT by four mutations within the RT sequence [14,20]. PFV, however, did not develop resistance to AZT, and the introduction of the SFVmac mutations into the PFV RT gene did not result in viruses resistant to the nucleoside inhibitor [20]. Regarding the high amino acid homology of the two enzymes, this result was not to be expected. In SFVmac, the mechanism of resistance is due to the removal of already incorporated AZT-monophosphate (AZTMP) in the presence of ATP and thus resembles that of HIV-1 RT [14,21,22].

It has been shown previously that retroviral PRs are only active as homodimers. To create the active center, each subunit of the homodimer contributes catalytic residues located in the conserved motif DT/SG [23]. However, SFVmac PR-RT behaves as a monomer in solution, but nevertheless exhibits PR activity. Catalytic PR activity could only be observed at NaCl concentrations of 2-3 M [15], indicating that hydrophobic interactions might promote dimerization. Furthermore, by prevalent methods the separately expressed 12.6 kDa PR domain was also found to be monomeric but active [15]. Only further analyses using NMR paramagnetic relaxation enhancement proved that transient, lowly populated dimers are being formed (Hartl MJ, Schweimer K, Reger MH, Schwarzinger S, Bodem J, Rösch P, Wöhrl BM: Formation of transient dimers by a retroviral protease, submitted). Contradicting results were obtained by gel filtration analysis with a purified C-terminally extended 18 kDa PR domain of PFV, which indicated that PFV PR might be dimeric [6].

To clarify these issues and to shed more light on the properties of SFVmac and PFV PR-RT, we set out to purify both enzymes from bacterial lysates and directly compare their secondary structure, oligomerization state, and activities.

## Results and Discussion

### Protein purification

Overexpression of PFV PR-RT in *E. coli *resulted in partial degradation by cellular proteases. Thus, we could not adopt the purification protocol established for SFVmac PR-RT [14]. Instead, we had to set up a new purification procedure for PFV PR-RT which includes Ni-affinity followed by hydrophobic interaction chromatography to remove the PR-RT degradation products. The yields were much lower than for SFVmac PR-RT. Nevertheless, pure soluble protein (> 95% purity, as judged from SDS-polyacrylamide gels) could be obtained.

### Biophysical properties

**Figure 1:**
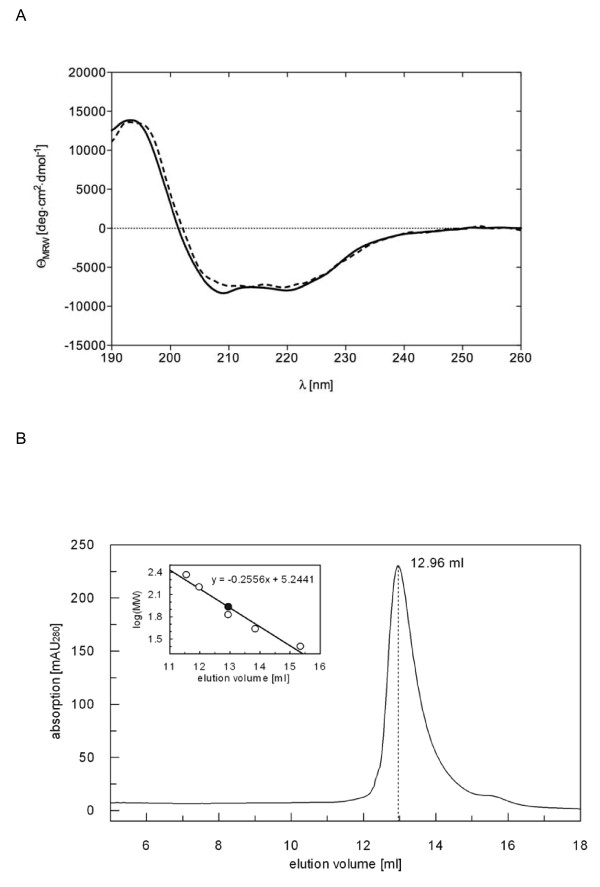
**Biophysical properties of PFV and SFVmac PR-RT**. **(A) **Far UV circular dichroism (CD) spectra of wild-type SFVmac (continuous line) and PFV PR-RT (dotted line) were acquired at 20°C using a band width of 1 nm, a sensitivity of 100 mdeg and a data density of 5 points/nm in a 0.1 cm cell with 0.5 μM of each enzyme in 25 mM Na_2_HPO_4_/NaH_2_PO_4 _pH 7.4, and 5 mM NaCl. **(B) **Size exclusion chromatography of PFV PR-RT using an S200 HR 10/30 column. The run was performed with 10 nmol PFV PR-RT in 50 mM Na_2_HPO_4_/NaH_2_PO_4 _pH 7.4, 300 mM NaCl and 0.5 mM DTT. The inset shows the fit to the data obtained for the molecular masses of the standard proteins (open circles), which was used for the determination of the molecular mass of PFV PR-RT (closed circle).

To exclude that the purified PR-RTs are partially or completely unfolded, we analyzed the secondary structure of PFV and SFVmac PR-RT by circular dichroism (CD) spectroscopy. The shape of the CD spectra obtained for the two enzymes was highly similar, implying comparable ratios of α-helices and β-strands (Fig. 1A). In both cases, the curves showed a broad minimum between 205 nm and 222 nm, characteristic for a mixture of α-helical and β-strand structures, and high ellipticity near 200 nm. Thus, the spectra are indicative of predominantly folded proteins. Although the spectrum obtained for SFVmac PR-RT deviates slightly from that of PFV PR-RT, the calculated values (Table 1) confirm the accordance in the secondary structure contents of PFV and SFVmac PR-RT. However, crystal structure analyses will be necessary to obtain more information on the structural similarities and differences of the two enzymes. The three-dimensional structure will probably also shed more light on the differences between PFV and SFVmac PR-RT in developing AZT-resistance.

**Table 1:** CD values

enzyme	α-helix (%)	β-sheet (%)	β-turns (%)	random coil (%)	total (%)
PFV PR-RT	22	30	20	27	99

SFVmac PR-RT	22	29	20	28	99

Contradicting data have been published on the monomer/dimer status of FV PRs. PFV PR expressed separately was suggested to be dimeric [6], whereas we have shown by various analyses, like size exclusion chromatography and analytical ultracentrifugation that the full length PR-RT protein as well as the separate PR domain of SFVmac are monomeric, and only transient PR dimers are being formed [15] (Hartl MJ, Schweimer K, Reger MH, Schwarzinger S, Bodem J, Rösch P, Wöhrl BM: Formation of transient dimers by a retroviral protease, submitted).

Previous results obtained by sucrose density gradient analyses with PR-RT purified from SFVmac particles also indicated that the protein is monomeric [24]. To clarify the monomer/dimer status of PFV PR-RT, we performed size exclusion chromatography (Fig. 1B). Our data revealed a single peak, which corresponded to a molecular mass of 85.4 kDa. This is in good agreement with the theoretical molecular mass of the monomeric PFV PR-RT of 86.5 kDa. Moreover, no dimer peak could be detected, indicating that under native conditions PFV PR-RT, like SFVmac PR-RT is monomeric to a great extent (> 95%).

### PR activity

Activity of retroviral PRs is only achieved when a symmetric homodimer is formed, since each subunit provides a conserved aspartate residue to form the active center [23,25,26]. To detect residual PR activity we used a substrate, denoted GB1-GFP, that consists of a fusion protein between the immunoglobulin binding domain B1 of the streptococcal protein G (GB1) and the green fluorescent protein (GFP) enframing the natural SFVmac Pol cleavage site YVVH↓CNTT. Although in PFV Pol the His is exchanged by Asn, this substrate could also be used for PFV PR-RT, because retroviral PRs are able to recognize different cleavage sites.

**Figure 2:**
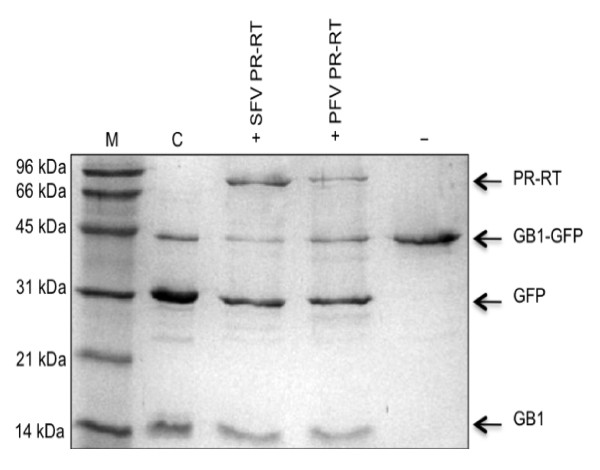
**PR activity assay**. Reaction products were analyzed by 19% SDS-PAGE. 10 μM GB1-GFP substrate harboring a FV PR cleavage site between GB1 and GFP was incubated with 10 μM SFVmac PR-RT or PFV PR-RT, respectively, at 37°C for 16 h in reaction buffer (50 mM Na_2_HPO_4_/NaH_2_PO_4 _pH 7.4, 0.5 mM DTT, 3 M NaCl). C, control, substrate cleavage with TEV protease; (-), uncleaved substrate; M, molecular weight standard. The sizes of the standard proteins are indicated on the left.

A concentration of 3 M NaCl was used in the assay since under these conditions SFVmac PR-RT revealed the highest PR activity, and no activity was detected when low salt concentrations (ca. 0.2 - 0.4 M NaCl) were applied [15]. Fig. 2 illustrates that both proteins were capable of almost completely cleaving the provided substrate even though the offered sequence is different from the naturally occurring cleavage site in PFV Pol.

Size exclusion chromatography and PR activity assays revealed a new feature special to *spumaretrovirinae*. FVs appear to express a monomeric PR domain within the Pol polyprotein which is catalytically inactive. *In vitro *dimerization of the PR domain is inducible at high salt concentrations. This effect might be caused by a hydrophobic dimerization interface, which under high ionic strength disfavors the monomeric state.

Recently published results suggest that HIV-1 PR in the Gag-Pol precursor is only present as a transient dimer due to an inhibitory effect of the transframe region, which is located N-terminally of the PR domain [27]. Since there is no Gag-Pol fusion protein in FVs, an N-terminal extension of the PR does not exist. Thus, the regulation of the FV PR activity has to be different. We have shown recently, that SFVmac PR forms transient dimers at low salt concentrations. Obviously, *in vivo *PR activation cannot be achieved by increasing the NaCl concentration to 3 M, indicating that an additional cellular and/or viral factor must be involved in PR activation.

### Characteristics of polymerization

A key step in the retroviral life cycle is the reverse transcription of the genomic RNA into double stranded (ds)DNA. For formation of dsDNA, the RT catalyzes RNA- and DNA-dependent DNA polymerization to synthesize the (-) and (+)-strand, respectively.

To further characterize the PR-RT enzymes, we performed polymerization assays on the homopolymeric poly(rA)/oligo(dT)_15 _substrate and on heteropolymeric single-stranded M13 DNA. The incorporation of ^3^H-TTP was used to determine Michaelis-Menten parameters. Comparison with values already published for SFVmac PR-RT for homopolymeric substrates revealed fairly similar K_M_- and k_cat_-values for the two enzymes. Moreover, the K_M_-values for homo- and heteropolymeric substrates are comparable (Table 2) [14].

**Table 2:** Kinetic parameters of the polymerization activities of SFVmac and PFV PR-RT

enzyme	K_D _DNA/RNA (nM)	K_D _DNA/DNA (nM)	K_M _^1)^ (TTP/rAdT) (μM)	k_cat_^1) ^(TTP/rAdT) (s^-1^)	K_M_^2)^(dNTPs/M13) (μM)	k_cat_^2) ^(dNTPs/M13) (s^-1^)
PFV PR-RT	9.9 (± 1.6)	44.4 (± 3.0)	45 (± 12)	7.1 (± 0.9)	46 (± 9)	3 (± 0.3)

SFVmac PR-RT	32.4 (± 4.2)^3)^	36.4 (± 2.4)^3)^	40.1 (± 4.0)^3)^	5.5 (± 0.3)^4)^	45 (± 3)	4 (± 0.1)

^1) ^K_M _and k_cat_-values, respectively, determined for TTP on the homopolymeric substrate poly(rA)/oligo(dT).

^2) ^K_M _and k_cat_-values, respectively, determined for dNTPs on a heteropolymeric single stranded M13 substrate

^3) ^Data adopted from [14]

^4) ^v_max_-value used for k_cat _calculation derived from [14].

The K_M _values determined here for FV PR-RTs are ca. 5-30 fold higher than those published for HIV-1 RT [28-30]. A recent publication compares the pre-steady-state kinetics of PFV PR-RT with those of HIV-1 and murine leukemia virus (MuLV) RT [31]. Although the k_pol _values of the three enzymes are similar, the dissociation constants (K_D_) for dNTP binding are about 10 - 80 fold higher with PFV PR-RT as compared to HIV-1 RT, but are comparable to the affinities obtained for MuLV RT [31]. These kinetic data together with our results reveal different polymerization properties of HIV-1 RT and FV PR-RTs. The data imply that DNA polymerization of FV PR-RTs is poor at low dNTP concentrations. One reason for the differences observed might be the fact that in contrast to FV, HIV-1 can replicate in non-dividing cells, where dNTP concentrations are low. In such an environment, polymerization efficiency can be improved by RTs with high affinities for dNTPs [31].

**Figure 3:**
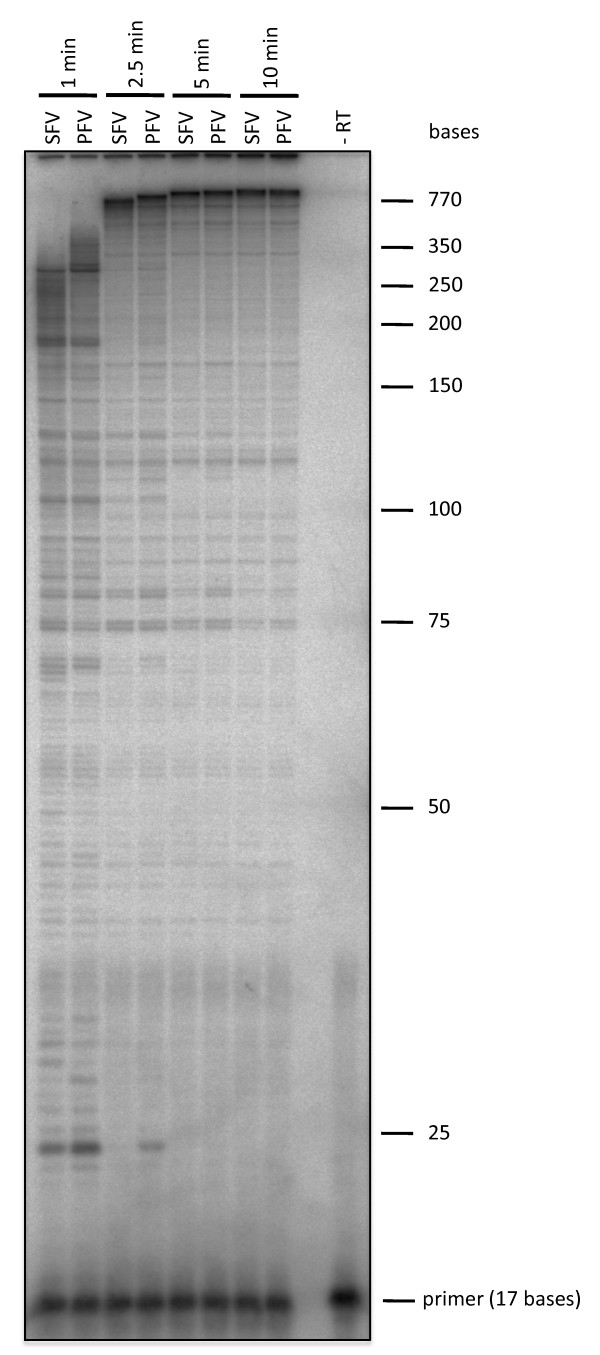
**DNA-dependent DNA polymerase activity on a heteropolymeric substrate**. Reactions were carried out at 37°C for the times indicated on top with 6 nM of the M13 P/T substrate, 85 nM of PFV or SFV PR-RT and 150 μM of each dNTP, analyzed by denaturing gel electrophoresis on a 10% sequencing gel and visualized by phosphoimaging. DNA size markers are marked on the right. - RT, assay without enzyme;

A qualitative analysis of DNA polymerization was performed by using a heteropolymeric single stranded M13 DNA as a template together with a radioactively 5' end labeled primer and saturating dNTP concentrations of 150 μM. The polymerization products were compared on a denaturing polyacrylamide/urea gel (Fig. 3). The results confirmed the kinetic data foreshadowed in Table 2, revealing a somewhat higher polymerization efficiency of PFV-PR-RT.

**Figure 4:**
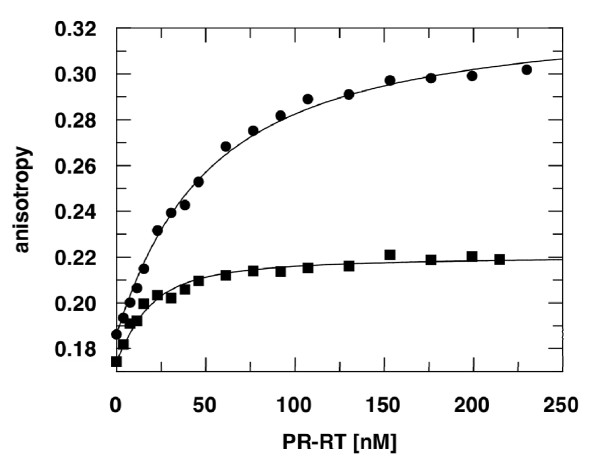
**Determination of K_D_-values by fluorescence anisotropy measurements**. 15 nM of a fluorescently labeled DNA/DNA (black circle) or DNA/RNA (black square) P/T substrate was titrated with PFV PR-RT at 25°C. The curves show the best fit to a two component binding equation [14] describing the binding equilibrium with K_D_-values shown in Table 2.

Since polymerization activities are also dependent on nucleic acid substrate affinities, we determined K_D_-values of the two FV PR-RTs for DNA/RNA and DNA/DNA by fluorescence anisotropy. In each of these experiments a 24/40 mer primer/template (P/T) substrate was used containing a fluorescent dye (Dy-647) at the 5' end of the template strand (Table 2, Fig. 4). For both enzymes, the affinity for the DNA/RNA P/T appeared to be higher than for DNA/DNA. This effect was far more pronounced for PFV PR-RT with a 4-fold lower K_D_-value for the DNA/RNA substrate. Comparison with HIV-1 RT shows an unexpected difference, i.e. the affinities of HIV-1 RT for nucleic acid substrates are much higher. For DNA/DNA or DNA/RNA substrates K_D_-values of approximately 2 nM have been determined [32-34].

### RNase H activity

The third enzymatic activity associated with PR-RT is its RNase H activity, which is responsible for degradation of the RNA strand of an RNA/DNA hybrid and is indispensable in the reverse transcription process.

Polymerization-independent RNase H activity was tested on two different substrates. First, Michaelis-Menten-parameters were determined on a blunt-ended RNA/DNA substrate containing a fluorescent dye on the 3' end of the RNA and a quencher on the 5' end of the DNA. Upon cleavage of the RNA the fluorescent dye is released from the quencher resulting in an increase in fluorescence intensity. By varying substrate concentrations, K_M_- and k_cat_-values for RNase H activities were calculated (Table 3). SFVmac and PFV PR-RT showed K_M_-values of 18.1 nM and 17.1 nM, respectively. These are in the range of HIV-1 RT (25 nM) [35] and *E. coli *RNase H (16 - 130 nM, depending on the substrate) [36]. Provided that indeed FV PR-RTs are less abundant in the virus particle, it is remarkable that the FV RNase H activities were not higher than those of HIV-1 RT.

**Table 3:** Kinetic parameters of the RNase H activities of SFVmac and PFV PR-RT

enzyme	K_M_ RNase H (nM)	k_cat _(s^-1^)
PFV PR-RT	17.1 (± 1.2)	0.017 (± 0.0003)

SFVmac PR-RT	18.1 (± 0.6)	0.020 (± 0.0003)

**Figure 5:**
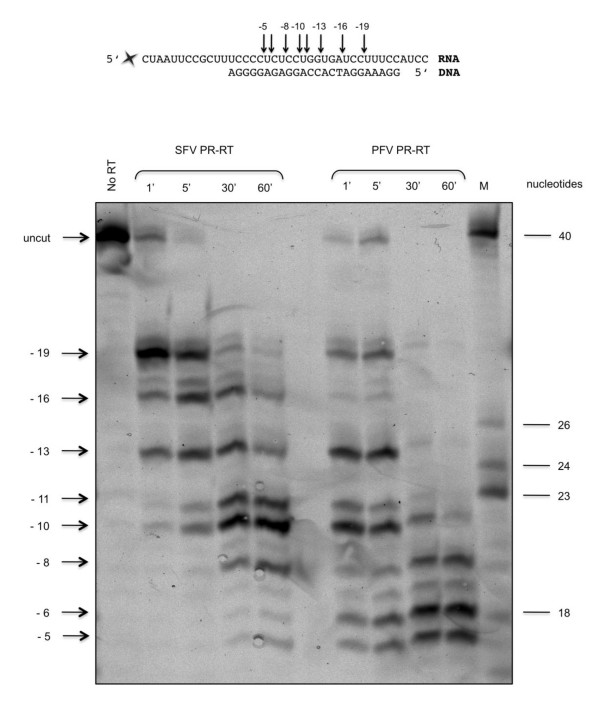
**Qualitative RNase H assay**. The DNA/RNA P/T substrate is shown on top. The cleavage sites determined for SFV and PFV PR-RT are indicated by arrows. 320 nM of DY-647 labeled P/T substrate was incubated with 50 nM of SFVmac or PFV PR-RT in 50 mM Tris/HCl, pH8.0, 80 mM KCl, 6 mM MgCl_2 _for the times indicated on top of the gel. Reaction products were analyzed on a 15% polyacrylamide sequencing gel and visualized by detection of the fluorescence emission of the RNA template strand at 670 nm upon excitation at 633 nm. The cleavage sites are indicated on the left. The first nucleotide of the RNA hybridized to the 3'-OH nucleotide of the DNA primer is denoted -1. The partially hydrolyzed RNA on the right was used for the determination of the cleavage sites. Numbers on the right indicate the length of the RNA in nucleotides.

To determine the endonucleolytic RNase H cleavage sites of the two PR-RTs qualitatively, a 40 mer RNA hybridized to a 24 mer DNA was used (Fig. 5). A fluorescent dye at the 5' end of the RNA allowed visualization of the cleavage products after separation on 15% sequencing gels. Our time course experiments indicated that with both enzymes a primary endonucleolytic cleavage at position -19 was followed by a 3' > 5' directed processing reaction leading to shorter RNA products (Fig. 5). Primary RNase H cleavage sites in the RNA at positions 15 -20 nucleotides away from the primer terminus of the hybrid were also detected for the RTs of *orthoretrovirinae *like HIV-1 and RSV [37-42]. They are directed by the 3'-end of the DNA-primer which binds to the active site of the polymerase [43,44]. While RSV RT appears to lack a 3' > 5' directed processing activity [37], SFVmac and PFV PR-RTs (Figure 5B) as well as HIV-1 and MoMLV RTs degrade the RNA to 8 mers or smaller products [41,45].

## Conclusions

Our data reveal small differences of FV PR-RTs in their catalytic activities and biophysical properties. The K_M_-values determined for HIV-1 RT are 5-30 fold lower than those for FV PR-RTs. These deviations in kinetic behavior might be based on the fact that HIV-1 can replicate in non-dividing cells. Remarkably, both FV PR-RTs are monomeric in solution, implying that transient dimers need to be formed in order to obtain PR activity. Transient dimerization has been demonstrated recently for SFVmac PR and was suggested to play a role in the regulation of a timely activation of PR activity (Hartl MJ, Schweimer K, Reger MH, Schwarzinger S, Bodem J, Rösch P, Wöhrl BM: Formation of transient dimers by a retroviral protease, submitted). Small structural and consequently catalytic variations between the two FV PR-RTs might account for the differences observed (e.g. in the resistance to the nucleoside inhibitor AZT.) Further structural and functional analyses will be necessary to elucidate these findings.

## Methods

### Plasmid construction and protein purification

For SFVmac PR-RT, gene expression and protein purification were performed as described previously [14]. The plasmid pET101TOPO-PFV-PR-RT-6His was constructed using the Champion™ pET Directional TOPO^® ^Expression kit (Invitrogen, Darmstadt, Germany). The N-terminus of the PFV PR-RT starts with the amino acids MNPLQLLQPL corresponding to the N-terminus of the PR gene. The C-terminus contains a 6 × His tag and exhibits the following amino acid sequence: ATQGSYVVNA-6His. The plasmid was transformed into the *Escherichia coli *(*E. coli*) strain BL21 (DE3) pREP4:GroESL [46], expressing *E. coli *chaperone proteins to facilitate folding of heterologous proteins. Cells were grown at 37°C in LB medium supplemented with 100 μg/ml ampicillin and 34 μg/ml kanamycin to an optical density of 600 nm (OD_600_) of ca. 0.8. The temperature was reduced to 16°C until an OD_600_of ca. 1.0 was reached. Expression of the recombinant PFV PR-RT-6His gene was then induced by the addition of 0.2 mM isopropyl-thiogalactoside (IPTG) at 16°C over night. Cells were harvested by centrifugation at 5000 g for 20 min at 4°C.

### Purification of SFVmac and PFV PR-RT

SFVmac PR-RT was purified as described previously [14]. PFV PR-RT was purified as follows by a combination of Ni-affinity and hydrophobic interaction chromatography:

#### Ni-NTA affinity chromatography

Cells were resuspended in 50 mM Na-phosphate pH 7.4, 300 mM NaCl, 10 mM imidazole, 0.5 mM dithiothreitol (DTT). After addition of lysozyme, DNase I and one protease inhibitor cocktail tablet (Complete, EDTA-free, Roche Diagnostics GmbH, Mannheim) the suspension was stirred on ice for 30 min. After cell lysis using a microfluidizer (Microfluidics, Newton, MA, USA) the suspension was centrifuged at 19100 g for 30 min at 4°C. Purification of the protein was performed by a step gradient applying increasing concentrations of up to 500 mM imidazole on a HisTrap column (HisTrap, GE Healthcare, München, Germany).

#### Hydrophobic interaction chromatography

Fractions containing PFV PR-RT were pooled and dialyzed (Spectra/Por, MWCO 50 000 Da) twice for at least 2 h against 50 mM Na-phosphate pH 7.4, 300 mM NaCl, 1 M (NH_4_)_2_SO_4 _and 0.5 mM DTT and then loaded onto a 5 ml butyl column (ButylFF, GE Healthcare, München, Germany). The protein was eluted by applying a step gradient from 1 M (NH_4_)_2_SO_4 _and 300 mM NaCl to 0 M (NH_4_)_2_SO_4 _and 0 M NaCl. After electrophoresis of the fractions on 10% SDS-polyacrylamide gels the relevant fractions were concentrated with Vivaspin concentrators (MWCO 10 000 Da) to a volume of 200 μl and dialyzed against 50 mM Na-phosphate pH 7.4, 100 mM NaCl 0.5 mM DTT.

Analyses using circular dichroism (CD) spectra and size exclusion chromatography were performed with freshly purified SFVmac and PFV PR-RT. For PR, polymerization and RNase H measurements the PFV PR-RT was dialyzed (Spectra/Por, MWCO 50 000 Da) against 50 mM Na-phosphate pH 7.4, 100 mM NaCl, 0.5 mM DTT and 15% glycerol over night, the glycerol concentration was then increased to 50% and the protein stored at -20°C.

### Peptide mass fingerprint (PMF) analysis

Protein bands of ca. 1 mm × 3 mm were excised from 10% SDS-polyacrylamide gels and the integrity and identity of PFV PR-RT was confirmed by peptide mass fingerprinting (ZMMK Köln, Zentrale Bioanalytik, Germany).

### Circular dichroism

Far UV circular dichroism (CD) spectra of wild-type SFVmac and PFV PR-RT were acquired at 20°C using a Jasco J-810 spectropolarimeter (Japan Spectroscopic, Gross-Umstadt, Germany) at a band width of 1 nm, a sensitivity of 100 mdeg and a data density of 5 points/nm in a 0.1 cm cell. 0.5 μM of each enzyme was measured in 25 mM Na-phosphate pH 7.4 and 5 mM NaCl. At least 12 scans in the range between 260 and 190 nm were averaged for each measurement, and the resulting spectrum was smoothed and normalized to a mean residual weight ellipticity [Θ_MRW_] (deg·cm^2^·dmol^-1^) using Jasco Spectra Manager Software. For secondary structure predictions based on the CD data the program CDSSTR (Dichroweb) [14,27] was used.

### Size exclusion chromatography

For analytical gel filtration of PFV PR-RT a Superdex 200 HR 10/30 column (GE Healthcare, Munich, Germany) calibrated with catalase (232 kDa), aldolase (158 kDa), ovalbumine (43 kDa) and chymotrypsinogen (25 kDa) (GE Healthcare, Munich, Germany) was used at a flow rate of 0.5 ml/min. The column was loaded with 10 nmol PFV PR-RT in 50 mM Na_2_HPO_4_/NaH_2_PO_4 _pH 7.4, 300 mM NaCl and 0.5 mM DTT.

### PR activity assay

PR activity was measured as described before using a substrate which contained the SFVmac Pol cleavage site ATQGSYVVH↓CNTTP that can also be used by PFV PR-RT. Control digests with TEV protease were performed with the same substrate since it harbors a TEV cleavage site adjacent to the FV PR cleavage site [15].

### Polymerization assays

RNA-dependent DNA polymerase activity was quantitated on a poly(rA)/oligo(dT)15 substrate (0.2 U/ml) (Roche Diagnostics GmbH, Mannheim, Germany) in a standard assay (30 μl reaction volume) as described previously [14]. For the determination of K_M_, v_max _and k_cat _values, reactions were performed with increasing concentrations of TTP of 25, 50, 75,125 or 250 μM. For the determination of kinetic parameters on a heteropolymeric substrate 100 nM of single stranded M13mp18 DNA and 15 nM of PR-RT was used. dNTP concentrations of 25, 50, 75, 125 and 250 μM were added, using [3H]-TTP (3000 Ci/mmol, Hartmann Analytic GmbH, Braunschweig, Germany) as a tracer. K_M_-values were calculated by linear regression using Eadie-Hofstee plots. k_cat _is defined as v_max_/enzyme concentration. Qualitative DNA polymerization assays on denaturing polyacrylamide/urea gels using single stranded M13mp18 DNA as a substrate were performed as described previously [14].

### Fluorescence anisotropy measurements

Fluorescence equilibrium titrations were performed to determine the dissociation constants (K_D_) for nucleic acid binding with a 24/40 mer DNA/DNA or DNA/RNA primer/template (P/T). Experiments and data fitting were carried out as described [14] with15 nM fluorescently labeled P/T at 25°C.

### RNase H activity assays

#### Substrate preparation

The RNA-strand 5'-CCG AUG GCU CUC CUG GUG AUC CUU UCC-6-FAM (6-carboxy-fluorescein) and the DNA-strand 5'-Dabcyl-GGA AAG GAT CAC CAG GAG AG were synthesized by biomers.net (Ulm, Germany). The hybrid was formed by mixing the two oligonucleotides at a ratio of 1:1.2 respectively in 20 mM Tris/HCl pH 8.0 and 20 mM NaCl, followed by heating at 95°C for 2 min and cooling down to room temperature over a time period of 2 h. The resulting substrate was stored in aliquots at -20°C.

#### RNase H enzyme kinetics

Steady-state fluorescence measurements were performed at 25°C on a Fluorolog-Tau-3 spetrofluorometer (HORIBA Jobin Yvon GmbH, Unterhaching, Germany). The assay was carried out in a total volume of 1.2 ml containing 50 mM Tris/HCl pH 8.0, 80 mM KCl, 6 mM MgCl_2 _and a final concentration of 1 nM PR-RT. To determine the Michaelis-Menten kinetic parameters the DNA-dabcyl/RNA-6-FAM P/T concentration was varied from 10 to 200 nM. Cleavage of the RNA in the hybrid leads to dissociation of a fluorescein labeled RNA fragment from the dabcyl quencher and thus to a fluorescence increase. Upon excitation of the substrate at 495 nm an increase in fluorescence emission can be detected at 520 nm. The maximum change in fluorescence intensity and thus complete substrate cleavage was determined by incubating the hybrid with a large excess of PR-RT (250 nM). Initial rates were calculated using the linear slope of the reaction progress curve where less than 5% of substrate was cleaved. Values for kinetic parameters (K_M _and v_max_) were obtained by linear Eadie-Hofstee regression of the Michaelis-Menten equation V_0 _= V_max_·[S_0_]/(K_m_+ [S_0_]). k_cat _is defined as v_max_/enzyme concentration.

#### Qualitative RNase H assay

The gelelectrophoretic assay used a 5' fluorescently labeled RNA-oligonucleotide (5'- [DY-647]-CUA AUU CCG CUU UCC CCU CUC CUG GUG AUC CUU UCC AUC C; biomers.net, Ulm, Germany), which was purified on a 20% denaturing polyacrylamide gel and then annealed to the unlabeled DNA-oligonucleotide 5'-GGA AAG GAT CAC CAG GAG AGG GGA (biomers.net, Ulm, Germany). The hybrid was formed by mixing 2 μM Dye647-RNA with 2.4 μM DNA primer in 20 mM Tris/HCl pH 8.0 and 20 mM NaCl, followed by heating at 95°C for 2 min and cooling at room temperature over a time period of 2 h. The RNase H reaction was performed at 37°C in a total volume of 30 μl in 50 mM Tris/HCl pH 8.0, 80 mM KCl and 6 mM MgCl_2 _with 320 nM P/T substrate. The reaction was initiated by the addition of 50 nM PR-RT. Aliquots were removed at different time points and analyzed by electrophoresis on a 15% polyacrylamide sequencing gel. Products were visualized by fluorescence emission at 670 nm upon excitation at 633 nm using a fluorescence laser scanner (FLA 3000, raytest, Straubenhardt, Germany).

## Abbreviations

CD: circular dichroism; *E. coli*: *Escherichia coli*; 6-FAM: 6-carboxy-fluorescein; GB1: immunoglobulin binding domain B1 of streptococcal protein G; GFP: green fluorescent protein; HIV-1: human immunodeficiency virus type 1; IPTG: isopropyl-thiogalactoside; LTR: long terminal repeat; MuLV: murine leukemia virus; PMF: peptide mass fingerprint; PFV: prototype foamy virus; SFVmac: simian foamy virus from macaques.

## Competing interests

The authors declare that they have no competing interests.

## Authors' contributions

BMW conceived and coordinated the study. MJH and FM performed the experiments, AR provided reagents and participated in designing the experiments. BMW and MJH wrote the paper. All authors read and approved the manuscript.
